# Within-species relationship of patchiness to both abundance and occupancy, as exemplified by seagrass macrobenthos

**DOI:** 10.1007/s00442-021-04985-w

**Published:** 2021-07-09

**Authors:** R. S. K. Barnes

**Affiliations:** 1 Department of Zoology and Entomology, Rhodes University, Makhanda, Eastern Cape, Republic of South Africa; 2 School of Biological Sciences and Centre for Marine Science, University of Queensland, Brisbane, QLD Australia; 3 Department of Zoology and Conservation Research Institute, University of Cambridge, Cambridge, UK

**Keywords:** Patchiness, Occupancy, Seagrass, Macrobenthos, Larvipary

## Abstract

For the first time, intraspecific relationships between the macroecological metrics patchiness (P) and both abundance (A) and occupancy (O) were investigated in a faunal assemblage. As a companion study to recent work on interspecific P, A and O patterns at the same localities, intraspecific patterns were documented within each of the more dominant invertebrates forming the seagrass macrobenthos of warm–temperate Knysna estuarine bay (South Africa) and of sub-tropical Moreton Bay (Australia). As displayed interspecifically, individual species showed strong A–O patterns (mean scaling coefficient − 0.76 and mean *R*^2^ > 0.8). All P–O relations were negative and most (67%) were statistically significant, although weaker (mean *R*^2^ 0.5) than A–O ones; most P–A ones were also negative but fewer (43%) achieved significance, and were even weaker (mean *R*^2^ 0.4); 33% of species showed no significant interrelations of either O or A with P. No species showed only a significant P–A relationship. Compared with interspecific P–A–O data from the same assemblages, power–law scaling exponents were equivalent, but R^2^ values were larger. Larviparous species comprised 70% of the total studied, but 94% of those displaying significant patchiness interrelationships; 5 of the 9 showing no P–A or P–O relationships, however, were also larviparous. At Knysna, though not in Moreton Bay, larviparous species also showed higher levels of occupancy than non-larviparous ones, whilst non-larviparous species showed higher levels of patchiness. Dominant Moreton Bay species, but not those at Knysna, exhibited homogeneously sloped P–O relationships.

**Supplementary Information:**

The online version contains supplementary material available at 10.1007/s00442-021-04985-w.

## Introduction

Neither resources nor organisms are dispersed evenly across space. In effectively all cases, some regions support concentrations of any given item whilst other regions are areas of scarcity. Areas of abundance occur across wide ranges of spatial scales, and may vary in their size, in distance from other such concentrations, and in the supported intensity or density of the items concerned. Differential levels of abundance per unit area are also well-known to be related to those of occupancy (i.e. frequency of occurrence), the more widely an organism is distributed the more abundant it is also likely to be (see Hansky 1982; Gaston et al. 2000, 2006). Indeed in a number of cases occupancy can be precisely estimated from knowledge of mean density and the associated variance (He and Gaston 2003; Gaston et al. 2006). This relationship is an important link between ecological pattern and process (Freckleton et al. 2005), and as such is potentially of considerable importance in understanding ecological assembly processes, species distribution patterns, and requirements for conservation of individual species and whole assemblages, including in the prediction of responses to such threats as global warming, habitat destruction, etc. (dos Anjos et al. 2011; Manne and Veit 2020).

Areas of high relative abundance may constitute patches and several widely used measures of patchiness (i.e. the degree of inequality of spatial distribution of abundance) are also based on expressions involving the same two metrics that may permit the accurate estimation of occupancy, mean density and the associated variance (e.g. Morisita 1959, 1962; Lloyd 1967). Recently the author sought to align such patchiness with the interspecific (i.e. between-species) macroecological abundance–occupancy relationship (Barnes 2019, 2021). A preliminary study of the interspecific relationship between patchiness, abundance and occupancy was conducted using the component macrobenthic species of the intertidal beds of seagrass, *Zostera capensis*, within the warm–temperate South African Knysna estuarine bay (Barnes 2019). This work was then broadened by investigating in comparable fashion the equivalent macrobenthic assemblages associated with intertidal beds of six other seagrass species (*Cymodocea serratula*, *Halodule uninervis*, *Halophila ovalis*, *Zostera muelleri* and *Z. noltei*) in sub-tropical Moreton Bay, Queensland, and on cool-temperate Scolt Head Island in the North Sea, together with subtidal beds of the *Z. capensis* at Knysna (Barnes 2021). Notwithstanding the great differences in overall macrofaunal abundance (> 60,000 to < 2000 ind m^−2^), overall species richness (> 200 to < 30), species composition, and habitat features of these assemblages, as well as their latitudinal and longitudinal separation, all showed a consistent interspecific relationship between patchiness, abundance and occupancy. The more abundant and widespread the species, the less was its patchiness, with a significant negative patchiness–occupancy relationship in the form of a power law with a mean scaling coefficient of − 0.76. Notwithstanding the close relationship of abundance and occupancy, that of patchiness to abundance, however, was less marked and not always statistically significant.

Abundance–occupancy relationships within individual species (i.e. intraspecific patterns) are seemingly less common and less uniform than interspecific ones (Gaston 1999; Gaston et al. 2001). Indeed Bijleveld et al. (2018) studying an equivalent assemblage of Wadden Sea macrobenthic species to those investigated by Barnes (2019, 2021) in Knysna and Moreton Bay, found many very poor abundance–occupancy relationships within various individual polychaete, gastropod and bivalve species. Such variation across species, however, has the potential to help understand causality of the abundance–occupancy relationship (Buckley and Freckleton 2010; Gaston et al. 2001; Freckleton et al. 2005; Verberk et al. 2010). As it may also illuminate relations with patchiness, this research revisits the databases underlying the earlier interspecific patchiness–abundance–occupancy analyses to present a corresponding investigation of the nature and magnitude of intraspecific relationships within the individual component species of those same seagrass assemblages.

## Methods

Fifty two datasets were available for analysis from two localities: (A) 26 detailing the abundance of the 12 most numerous and widespread individual component species of the seagrass macrobenthos from apparently uniform (i.e. non-patchy) local areas of seagrass (each < 1 ha in size) along the Rainbow Channel coast of North Stradbroke Island (Minjerribah) in the Moreton Bay (Quandamooka) Marine Park, Queensland, Australia; and (B) the same number containing similar information for the 15 equivalent species in non-fragmented seagrass beds in the Knysna estuarine bay, Garden Route National Park, Western Cape, South Africa (see Electronic Supplementary Information 1). All datasets from each locality were located within a linear distance of 7 km, and were from a single uninterrupted expanse of coastal seagrass across which dispersal throughout the whole locality was possible. The qualification for inclusion of a species was its presence in at least ten datasets in abundances of > 35 m^−2^ in low-density Moreton Bay or of > 80 m^−2^ in higher-density Knysna, and occurrences of > 1 ind. in any single core sample in all qualifying datasets. Analysis was therefore confined to relatively common species, not only because confidence in nature of dispersion assessed from relatively few samples is low (Green 1966) but also because abundance–occupancy relationships may be different in rare versus widespread species (Borregaard and Rahbek 2006; Bijleveld et al. 2018), regression slopes being shallower and coefficients of determination (*R*^2^) weaker in the rare category (Freckleton et al. 2006; Buckley and Freckleton 2010). Although the intertidal and immediately subtidal shores of the two localities were both dominated by continuous swards of dwarf-eelgrasses (*Zostera* subgenus *Zosterella* = *Nanozostera* in the revision of Coyer et al. 2013), there were differences between them. At Knysna, shores varied widely in form, from extensive, tide-washed flats of marine sands at the mouth to steeply sloping banks of soft estuarine mud at the head, whilst those in Moreton Bay were much more uniform, extensive mangrove-backed flats.

Sampling of these two seagrass macrobenthic assemblages used the same methodology, involving series of core samples (0.0054 m^2^ area; 100 mm depth), with a minimum 30 cores per dataset at Knysna and 45 in Moreton Bay. Intertidal samples were collected at low tide before complete tidal ebb whilst the substratum was still covered by at least 15 cm of water, and the subtidal ones (some 1.5 m below low water spring level) by snorkelling. Subtidal and intertidal assemblages differed quantitatively but not qualitatively (Barnes and Claassens 2020). Cores were gently sieved ('puddled') through 710 µm mesh on site. This sampling procedure collects the smaller (mostly < 5 mm) and more numerous members of the macrofauna that constitute the large majority of invertebrate biodiversity (Bouchet et al. 2002; Albano et al. 2011), though not the meiofauna nor much scarcer megafauna nor sessile animals attached to the seagrass leaves. Warwick et al. (2006) have shown that different patterning rules may apply to meiofauna and macrofauna, and likewise Davidson et al. (2004) and Leopardas et al. (2014) to sessile species.

Retained material from each core was: (1) placed in a large container of local sea water within which all seagrass was shaken vigorously to dislodge all but sessile animals; (2) then re-sieved and transported immediately to a local laboratory, and (3) there placed in a 30 × 25 cm tray over a light source in which the living fauna was located by visual examination using 3.5× magnifying spectacles until no further animal could be observed. Animals were identified to species level wherever possible, with all organismal nomenclature here being as listed in the World Register of Marine Species (www.marinespecies.org) (accessed November 2020), except for the currently genus-less '*Assiminea*' *capensis* (see Barnes 2017). It should be noted, however, that the specific identity of several of the animals is questionable, especially amongst the Polychaeta and Peracarida, because of lack of relevant systematic studies in the geographical regions concerned*.* Such animals were treated as morphospecies, an operationally appropriate procedure to detect spatial patterns in numbers of species and their differential abundance (Dethier and Schoch 2006; Gerwing et al. 2020).

All abundance data are presented as densities (numbers m^−2^) and for individual species calculation of mean densities included unoccupied samples (i.e. zero values), occupancies being proportions of the total samples in a given dataset in which a species was present. In conformity with the interspecific data presented earlier (Barnes 2019, 2021), magnitude of patchiness was ascertained by spatial point pattern analysis of count data using Lloyd's index of patchiness (Lloyd 1967), *I*_p_ = [1 + 1/*k*], where *k* is the dispersion parameter of the negative binomial distribution, i.e. = [1 + (v−m)/m^2^], where ‘*m*’ is the mean abundance across samples and ‘*v*’ is the associated spatial variance. This index has been demonstrated to yield equivalent results to those of the spatially-explicit Moran's spatial auto-correlation index for intertidal dwarf-eelgrass macrobenthos (Barnes and Hamylton 2019). It is also independent of sample size over a wide range of areas, provided that the animals position themselves at random with respect to each other within a patch and that the patches are large relative to sample size (Lloyd 1967; Myers 1978). Granted that individual core area was 0.0054 m^2^, it seems unlikely that macrofaunal patches were smaller than that; indeed Barnes (2016) had previously found at a Knysna seagrass locality that cores of 0.0015, 0.0026 and 0.0054 m^2^ spatial grain all produced the same value of the closely-similar but differently-derived Morisita's *I*_δ_ index. Patchiness of dispersion of individual patches was assessed from geo-referenced samples by nearest-neighbour analysis.

All calculations and analyses were carried out in Microsoft Excel for Mac 16.37 with the StatPlus:mac Pro 7.1.1 add-on, or via *PAST* 3.24 (Hammer et al. 2019). Statistical comparisons were effected by ANOVA, ANCOVA, Mann–Whitney *U* tests, and Spearman rank correlation (*S*_r_), as appropriate; and curves were fitted using KaleidaGraph 4.5.4. To enable direct comparison with the earlier interspecific results (Barnes 2019, 2021), the present intraspecific occupancy data are also given in log percentage occurrence form rather than as logit transformations: transformation of occupancy values has no effect on either rank correlation between occupancy and patchiness (or abundance) or on the pattern of relative position of individual data points across logarithmic space (and values of *R*^2^ were not significantly different: ANOVA *F*_1,16_ = 0.86; *P* = 0.37). Occupancy data were, however, logit transformed to test homogeneity of slopes of the occupancy–patchiness relationship by ANCOVA. Power–law scaling coefficients (exponents) are abbreviated below to *β* and their coefficients of determination to *R*^2^. Information on the juvenile forms of the various species was derived from the available literature.

## Results

**Fig. 1:**
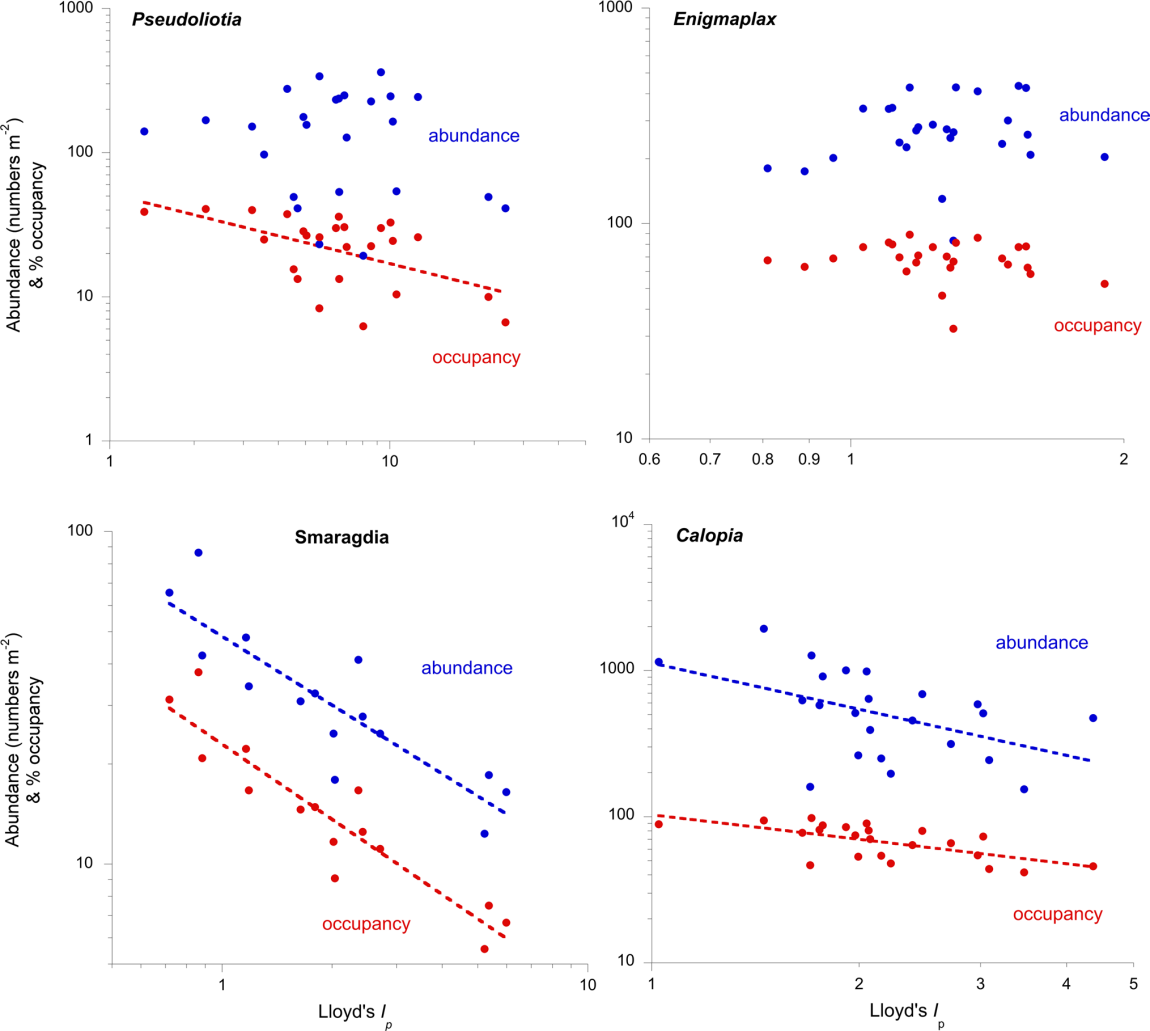
Intraspecific patchiness–abundance and patchiness–occupancy relationships in the seagrass macrobenthos of the Rainbow Channel shores of North Stradbroke Island (Minjerribah), Moreton Bay (Quandamooka), Queensland, illustrated by four species representing the variety of responses shown. Power law curves are indicated only when correlations between metrics are significant at *P* < 0.05

**Fig. 2:**
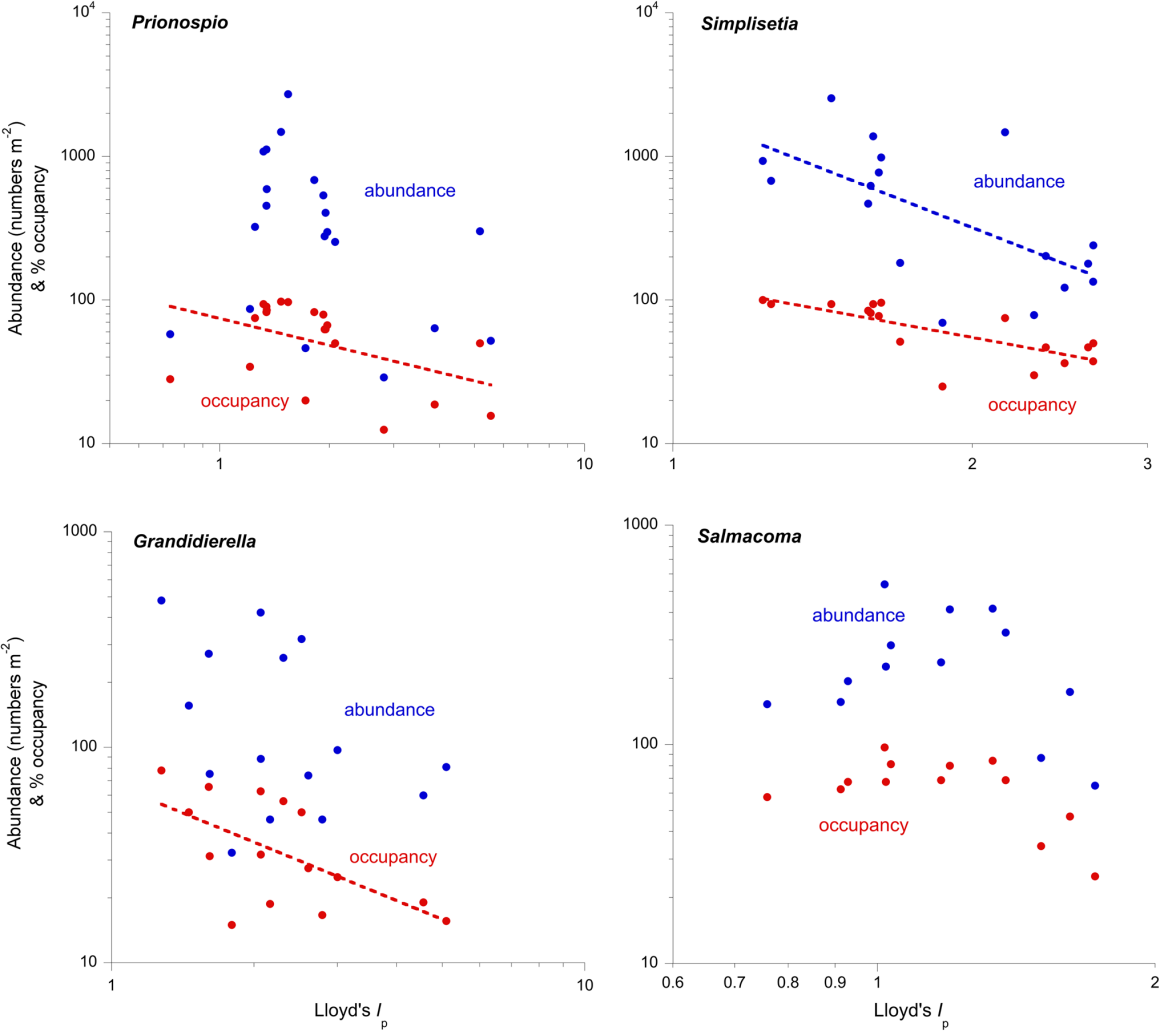
Intraspecific patchiness–abundance and patchiness–occupancy relationships in the seagrass macrobenthos of the Knysna estuarine bay, South Africa, illustrated by four species representing the variety of responses shown. Power law curves are indicated only when correlations between metrics are significant at *P* < 0.05

All relationships between patchiness and occupancy in the individual species under study were negative, although only 18 of them (nine at each locality) were significant at *P* < 0.05. Most (85%) of those between patchiness and abundance were also negative, although only ten of them were significantly so (five at each locality) (Tables 1 and 2). Individual species referred to below are listed in full in Tables 1 and 2, but are here referred to only by their genera for simplicity since each such genus was represented only by a single species in the group of dominants. Species varied widely from those displaying a significant negative correlation of patchiness with both metrics, e.g. *Simplisetia*, *Danielella*, '*Assiminea*', *Nassarius* and *Arcuatula* at Knysna, and *Malacoceros*, *Alpheus* and *Smaragdia* in Moreton Bay; through a significant negative correlation only with occupancy (*Prionospio*, *Pseudofabricia*, *Exosphaeroma* and *Grandidierella* at Knysna, and *Pseudoliotia*, *Tritia* and *Dasybranchus* in Moreton Bay); to no correlation with either (six species at Knysna including *Caulleriella*, *Cymadusa* and *Alaba*, and *Eriopisella*, *Longiflagrum* and *Enigmaplax* in Moreton Bay) (Figs. 1 and 2). No species displayed a correlation only between patchiness and abundance; and in no geo-referenced series of samples did the occupied cores themselves display a patchy dispersion (all nearest neighbour *R*_n_ > 1.1). All ten species that showed a significant correlation of patchiness with both abundance and occupancy are (or, where such information is not available, could reasonably be assumed to be) larviparous, notwithstanding that direct developers comprised 30% of the species investigated. Further, all but one species that only showed a significant relationship of patchiness with occupancy were also larviparous. Nevertheless, across the whole dataset there was no significant difference between larviparous and non-larviparous species in their patchiness, abundance or occupancy, or in their abundance–occupancy relationship (either *β* or *R*^2^) (ANOVA *F*_1,25_ < 1.9; *P* > 0.2). Otherwise the three categories of response above comprised a cross section of infaunal/epifaunal species and those of the represented higher taxa.

**Table 1:** Intraspecific patchiness–abundance (P–A) and patchiness–occupancy (P–O) relationships of dominant components of seagrass macrofaunal assemblages in the Knysna estuarine bay (Western Cape, South Africa)

	P	A	P–A *S*_r_	Sign	*β*	*R*^2^	O	P–O *S*_r_	Sign	*β*	*R*^2^	*n*
*Pseudofabricia capensis* (sabellidan polychaete)												
	4.25	186	− 0.52	0.1			28	− 0.72	0.02	− 0.56	0.43	10
*Exosphaeroma hylecoetes* (isopod crustacean)												
	4.08	84	− 0.40	0.1			27	− 0.66	0.01	− 0.62	0.37	15
*Caulleriella capensis* (cirratuliform polychaete)												
	3.87	385	− 0.25	0.4			50	− 0.44	0.2			11
*Nassarius kraussianus* (buccinoid gastropod)												
	3.55	373	− 0.79	0.0001	− 1.13	0.59	51	− 0.88	0.0001	− 0.78	0.86	23
*Paradoneis lyra capensis* (paraonid polychaete)												
	3.44	259	− 0.22	0.4			39	− 0.31	0.3			12
*Alaba pinnae* (cerithioid gastropod)												
	3.28	4211	− 0.33	0.2			64	− 0.41	0.1			14
*Danielella edwardsii* (brachyuran crustacean)												
	2.70	98	− 0.58	0.04	− 1.01	0.22	32	− 0.68	0.01	− 1.04	0.43	13
'*Assiminea*' *capensis* (truncatelloid gastropod)												
	3.11	543	− 0.88	0.0002	− 3.15	0.46	47	− 0.92	0.0001	− 1.73	0.80	12
*Grandidierella* sp. (amphipod crustacean)												
	2.46	167	− 0.39	0.1			38	− 0.60	0.02	− 0.90	0.41	15
*Arcuatula capensis* (mytiloid bivalve)												
	2.41	314	− 0.68	0.003	− 1.36	0.24	56	− 0.74	0.001	− 1.10	0.64	17
*Cymadusa* c.f. *filosa* (amphipod crustacean)												
	2.29	85	− 0.39	0.2			29	− 0.62	0.05			10
*Prionospio sexoculata* (spionidan polychaete)												
	2.12	543	− 0.37	0.1			60	− 0.47	0.04	− 0.62	0.12	20
*Simplisetia erythraeensis* (nereid polychaete)												
	1.92	651	− 0.61	0.01	− 1.05	0.33	66	− 0.79	0.0002	− 0.55	0.40	17
*Dosinia hepatica* (veneroid bivalve)												
	1.59	125	0.10	0.7			39	− 0.03	0.90			10
*Salmacoma littoralis* (tellinoid bivalve)												
	1.15	243	0.01	0.9			64	− 0.18	0.50			14
Mean			− 0.42		− 1.54	0.37		− 0.56		− 0.88	0.50	

Species are listed in descending order of patchiness

*P*  mean Lloyd's index of patchiness, *A* mean abundance m^−2^, *S*_*r*_ correlation coefficient,*Sign* probability of *S*_r_ value, *β* scaling coefficient of fitted power law, *R*^2^ coefficient of determination of fitted power law, *O*  mean percentage occupancy, *n*  number of datasets

**Table 2:** Intraspecific patchiness–abundance (P–A) and patchiness–occupancy (P–O) relationships of dominant components of seagrass macrofaunal assemblages in Moreton Bay (Queensland, Australia)

	P	A	P–A *S*_r_	Sign	*β*	*R*^2^	O	P–O *S*_r_	Sign	*β*	*R*^2^	*n*
*Pseudoliotia speciosa* (truncatelloid gastropod)												
	7.84	157	0.01	0.9			24	− 0.51	0.01	− 0.49	0.32	25
*Eriopisella moretoni* (amphipod crustacean)												
	4.44	81	− 0.02	0.9			24	− 0.35	0.3			10
*Longiflagrum caeruleus* (tanaid crustacean)												
	3.73	140	− 0.08	0.7			32	− 0.40	0.1			17
*Armandia* c.f. *lanceolata* (ophelioid polychaete)												
	3.64	68	− 0.66	0.04	− 0.55	0.43	24	− 0.83	0.003	− 0.71	0.67	10
*Tritia burchardi* (buccinoid gastropod)												
	3.53	73	− 0.37	0.1			25	− 0.59	0.008	− 0.75	0.35	19
*Malacoceros* ?*reductus* (spionidan polychaete)												
	2.89	94	− 0.61	0.04	− 0.76	0.28	34	− 0.61	0.04	− 0.87	0.43	11
*Dasybranchus caducus* (capitelloid polychaete)												
	2.52	71	− 0.42	0.1			26	− 0.72	0.006	− 0.91	0.46	13
*Smaragdia souverbiana* (neritoid gastropod)												
	2.42	35	− 0.86	0.0001	− 0.69	0.71	16	− 0.91	0.0001	− 0.75	0.81	15
*Calopia imitata* (truncatelloid gastropod)												
	2.26	622	− 0.54	0.01	− 1.05	0.33	70	− 0.69	0.0005	− 0.56	0.40	23
*Alpheus papillosus* (macruran crustacean)												
	2.17	58	− 0.64	0.02	− 0.68	0.49	25	− 0.70	0.007	− 0.77	0.60	13
*Limnoporeia* c.f. *yarrague* (amphipod crustacean)												
	1.89	159	− 0.11	0.6			45	− 0.52	0.03	− 0.49	0.23	19
*Enigmaplax littoralis* (brachyuran crustacean)												
	1.28	278	0.17	0.4			68	− 0.21	0.30			26
Mean			− 0.35		− 0.75	0.45		− 0.59		− 0.70	0.47	

Species are listed in descending order of patchiness

*P* mean Lloyd's index of patchiness, *A* mean abundance m^−2^, *S*_r_ correlation coefficient, *Sign* probability of *S*_r_ value, *β* scaling coefficient of fitted power law, *R*^2^ coefficient of determination of fitted power law, *O*  mean percentage occupancy, *n*  number of datasets

Few significant correlations occurred between individual metrics and such life-style features, although at Knysna direct-developers showed higher levels of patchiness than larviparous forms (one-way ANOVA *F*_1,13_ = 3.91; one-sided *P* = 0.035), whilst larviparous species showed higher levels of occupancy than direct developers (one-way ANOVA *F*_1,13_ = 6.96; one-sided *P* = 0.01). In Moreton Bay, those species that displayed a significant relationship between patchiness and abundance were all larviparous. Difference between those species showing a relationship between patchiness and occupancy (or both occupancy and abundance) and those showing no such relationship was not solely a matter of developmental mode. Larviparous species such as those of *Paradoneis*, *Alaba*, *Dosinia* and *Salmacoma* in Knysna, and *Enigmaplax* in Moreton Bay, also showed no relationship of patchiness with either metric. *Salmacoma* and *Dosinia*, at least, have similar local autecologies, and tend to occur in the same habitat type; they also show a significant correlation of their local abundance (e.g. *S*_r_ = 0.30 and *P* = 0.0001 in 2019 data and *S*_r_ = 0.22, *P* = 0.0001 in 2020). Although there was no significant relationship between patchiness and abundance (*S*_r_ = − 0.20; *P* = 0.3), there was a significant negative correlation between abundance and the extent to which local patchiness of a species varied, as assessed by its coefficient of variation (*S*_r_ = − 0.62; *P* = 0.03).

**Fig. 3:**
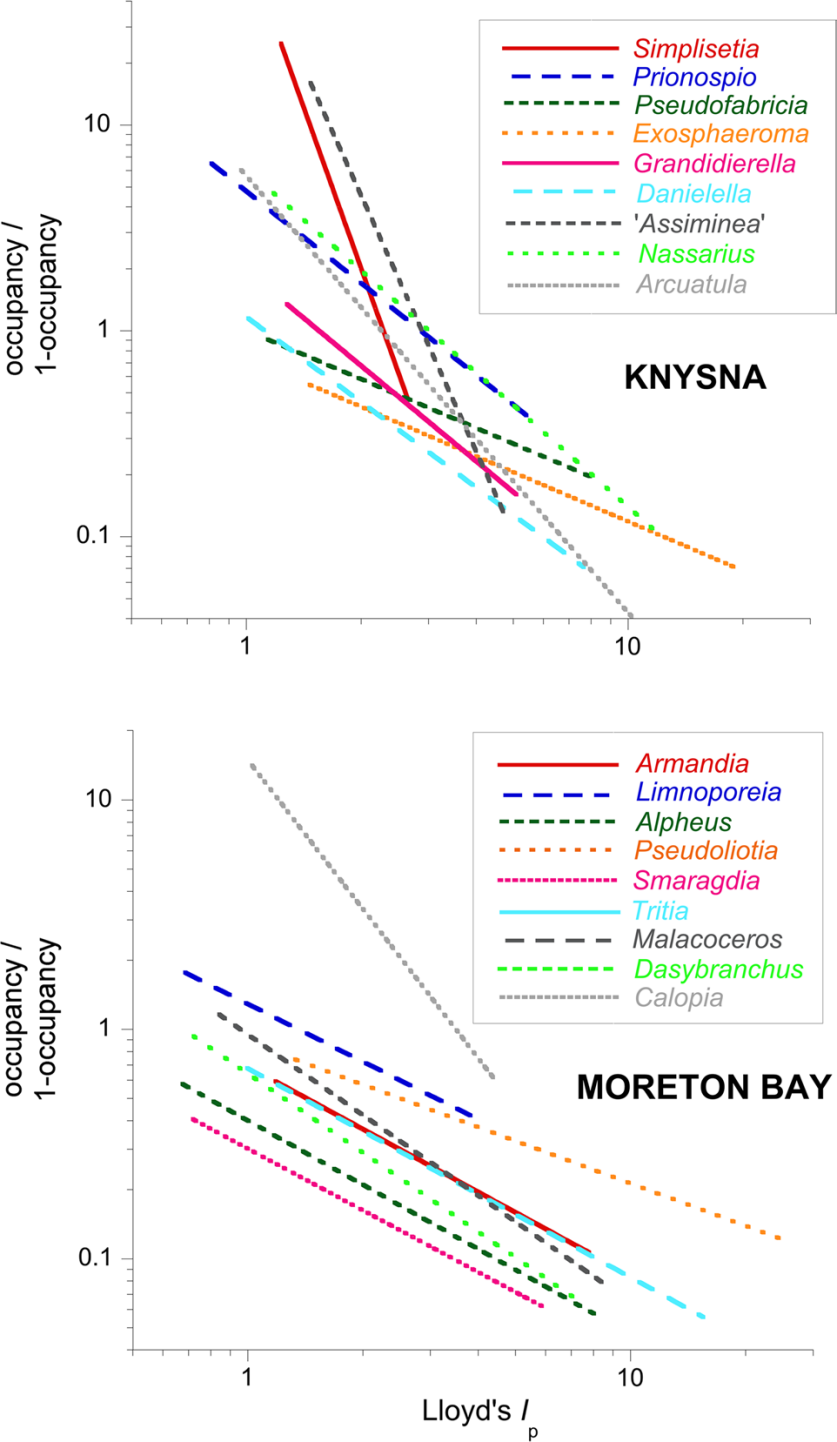
Power–law slopes of significant intraspecific occupancy–patchiness relationships amongst dominant members of seagrass macrobenthos: heterogeneous across the disparate Knysna estuarine-bay meadows and homogeneous in relatively uniform Moreton Bay

Relationships between abundance and occupancy were strong in both localities: Knysna mean species *S*_r_ = 0.919 (SD 0.053); all *P* < 0.003; Moreton Bay mean *S*_r_ = 0.881 (SD 0.10); all *P* < 0.003, with high values of R^2^ for the power–law curves (a mean of 0.81 at Knysna and of 0.82 in Moreton Bay). The log abundance—log occupancy relationships in Moreton Bay were of the standard linear form O = *αA*^*β*^, whilst most of those at Knysna were curvilinear, i.e. O = *γ* + *αA*^*β*^. The relationships of each metric with log patchiness were much weaker (mean values of *R*^2^ of 0.37 and 0.45 for significant patchiness and abundance relationships at Knysna and Moreton Bay, and 0.50 and 0.47 for the corresponding significant relationships with occupancy at those localities). Slopes of the power law curves for abundance versus patchiness at both localities varied across species (ANCOVA *F* =  > 4.24; *P* =  < 0.004), as did those of logit occupancy versus log patchiness at Knysna (ANCOVA *F* = 4.9; *P* =  < 0.0001); but those for the logit occupancy—log patchiness relationship were homogeneously sloped in Moreton Bay species (ANCOVA *F* = 1.48; *P* = 0.17) (Fig. 3), at a mean *β* of − 0.93 (SE 0.06) excluding the relatively steeply-sloped *Calopia*. There were no significant differences in values of *β* or *R*^2^ between the Knysna and Moreton Bay species (ANOVA *F* < 3.7; *P* > 0.1). Comparing the interspecific power law relationships of Barnes (2021) with the present intraspecific ones, there were no significant differences between the *β* values of either the abundance or occupancy relationships with patchiness (Mann–Whitney *Z* < 1.6; *P* > 0.1), but *R*^2^ values were larger in the present intraspecific curves both for patchiness–abundance and patchiness–occupancy (*Z* > 2.7; *P* < 0.006).

## Discussion

Clearly, it is likely that some form of relationship occurs between, on the one hand, the spatial dispersion of individual organisms (whether of the same or of different species) and, on the other, occupancy–abundance patterns (Gaston et al. 1998a, b; Falster et al. 2001; Holt et al. 2002), not least because occupancy is itself a spatial phenomenon and abundance is never distributed evenly across space (except intraspecifically over very limited areas in a few cases). Indeed, in their review of various spatial statistical models that describe and might possibly explain the interspecific occupancy–abundance relationship, Holt et al. (2002) included ones based on the negative binomial that also forms part of expressions such as Lloyd’s *I*_p_ measure of patchiness. They did not include patchiness models in their analyses, however, presumably because patchiness can never be an explanation for occupancy–abundance patterns: as above, it is a measure of the uneven spatial distribution of abundance, whereas, the occupancy–abundance relationship is based on co-variation of mean values of those two metrics.

The present study yielded information on the intraspecific patchiness–abundance–occupancy relationships within each of the more dominant component species that together partly comprised the macrofaunal seagrass assemblages of Moreton Bay and Knysna. Comparison of the values obtained with those of the equivalent interspecific relationships at those localities documented earlier (Barnes 2021) suggests that the intra- and interspecific relationships are very similar. In both cases, all patchiness–occupancy relations were negative and most correlations between the two were statistically significant. Patchiness and abundance, on the other hand, were both more poorly correlated and the relationship was often not significant. The mean interspecific and intraspecific values of *R*^2^ for the abundance–occupancy relationship were comparable and at the high end of the range quoted by Gaston (1996) (0.89 interspecifically and 0.81 intraspecifically), whilst no significant differences between the levels of intra- and interspecific *β* for the patchiness–abundance or patchiness–occupancy relationships were detected. The only general difference of note was that the *R*^2^ values for those relationships were larger intraspecifically. Within the intraspecific series of responses, however, the parallel nature of slopes for the power-law relationship between patchiness and occupancy across the dominant species in the Moreton Bay seagrass is worthy of particular comment, not least because it contrasts with that observed at Knysna. The habitats investigated in Moreton Bay were much more uniform and homogeneous than those at Knysna, and it does suggest a potential commonality of response to the same type of conditions across a wide range of different species exhibiting varying levels of occupancy and different life styles and dispersal strategies.

This study appears to be the first in which a comparison of intra- and interspecific metrics deriving from the same faunal assemblages has been undertaken, and certainly the first to involve relationships with levels of patchiness. As such, it is of course difficult to place these results in a wider context of patchiness research. Some other studies, however, have concerned abundance and occupancy in the marine environment, where Blackburn et al. (2006) considered the strongest abundance–occupancy relationships to be found. Indeed two have investigated similar soft-sediment estuarine or estuarine-bay habitats. Across a range of British estuarine invertebrates, Foggo et al. (2003) found an overall value of the interspecific *β* of 0.58 (and a range of 0.44–0.87 for various higher taxa), and an overall R^2^ value of 0.64 (0.55–0.75 across higher taxa). The equivalent overall interspecific abundance–occupancy *β* and the value of *R*^2^ at the present localities [a *β* of 0.67 (SE 0.04) and *R*^2^ of 0.81 (SE 0.03)] were larger than those of Foggo et al. (2003), but nevertheless, were of the same general order of magnitude. They are much larger on average and much more uniform, however, than those obtained by Bijleveld et al. (2018). These authors recorded values of *R*^2^ for the occupancy–abundance relationship of some individual macrobenthos in the Netherlands Wadden Sea as low as 0.00 and 0.01 (i.e. in *Abra tenuis*, *Macomangulus tenuis* and *Nephtys hombergi*), and generally found only weak relationships with median values of *β* in the range of − 0.03 to 0.33. It should be noted, however, that Bijleveld et al. (2018) quoted the values of *β* and *R*^2^ for ‘relationships’ even when no significant relationship was present. Here, such values are solely presented for significantly correlated metrics, and so any contrast in patterns between the Wadden Sea and the southern hemisphere localities may be more apparent than real: present values of *R*^2^ where no relationship occurred would also have varied down to 0.002 (P–A) and 0.03 (P–O). The results of Bijleveld et al. (2018) also dramatically emphasise the greater variation generally observed in individual intraspecific relationships than in interspecific ones, a position with which the present results also conform: two thirds of the individual species displayed significant patchiness–occupancy relationships; c.f. all but one of the interspecific assemblages of Barnes (2021) and that one exception was very close to significance at *P* = 0.061. But, as noted above, although individual species may differ considerably, their datapoints lay closer to the power law regressions than was the case interspecifically.

Freckleton et al. (2005), Foggo et al. (2007), Webb et al. (2009) and others have shown a relationship between developmental mode and abundance–occupancy patterns, larviparous species with consequent high dispersal rates showing the stronger and steeper relationships. No such general pattern was evident in the present results, although relatively little is known of the precise mode and dispersal potential of most of the species under study. However, there were marked effects of larvipary versus non-larvipary on patchiness–occupancy. Effectively all species across both localities (with the exception only of the amphipod *Limnoporeia* in Moreton Bay) showing a significant relationship of patchiness with occupancy were larviparous, and the directly-developing *Limnoporeia* displayed the lowest significant value of *R*^2^ of any species in the Bay (0.23). Whether the effect is caused by dispersal potential is less obvious because the non-larviparous species (mostly peracaridan crustaceans), and certainly those in South Africa, are very widely dispersed and characteristic members of the coastal/estuarine fauna (Schlacher and Wooldridge 1966; Henniger and Froneman 2011), tidal water fluxes in the habitats concerned are large, and the distances between samples were small. Small cores such as those used in the study may underestimate levels of occupancy (Lyashevska et al. 2016) but that should not differentially affect the non-larviparous.

Not all the species that failed to show a significant relationship between patchiness and either abundance or occupancy were non-larviparous, however; *Enigmaplax* in Moreton Bay, and *Paradoneis*, *Alaba*, *Dosinia* and *Salmacoma* at Knysna are all larviparous. No other feature appears to unite these species and hence their status is currently inexplicable, although the small group does include two of the most numerous species, *Alaba* at Knysna and *Enigmaplax* in Moreton Bay, the latter species in particular with a consistently high and therefore relatively limited range in level of occupancy. Barnes (2020) has noted that the most abundant species in Moreton Bay seagrass showed the greatest levels of uniformity in values of local patchiness, and this is confirmed by the present study in which there was a significant negative correlation between abundance and the extent to which patchiness of a species varied. Granted that there is so little information yet available, however, both in terms of the distribution of patchiness across the component species of an assemblage and of how those patchinesses relate to other macroecological variables, a general lack of understanding is hardly surprising. But although many uncertainties remain, the present intraspecific results together with the earlier interspecific ones do suggest that at least in the seagrass habitat, and probably elsewhere, patchiness is characteristically negatively related to levels of occupancy and, to a lesser degree, of abundance both within and across species. Granted the known ranges and median values of *R*^2^ for (a) the abundance–occupancy relationship and (b) now for that between patchiness and at least occupancy, confidence must be high that patchiness can be successfully integrated into what has been considered the most robust, pervasive, ubiquitous, and well documented pattern in macroecology (Verberk et al. 2010; Roney et al. 2015). This can only increase its usefulness.

## Supplementary Information

Below is the link to the electronic supplementary material.

Supplementary file1 (DOCX 11544 kb)
