# Supplementary material for: Within-species relationship of patchiness to both abundance and occupancy, as exemplified by seagrass macrobenthos
Source: Oecologia. 2021 Jul 9;196(4):1107–17. doi: 10.1007/s00442-021-04985-w (PMC8367887; doi:10.1007/s00442-021-04985-w)
Supplement: Supplementary file 1 — Supplementary file1 (DOCX 11544 kb) [file 442_2021_4985_MOESM1_ESM.docx]

**Within-species relationship of patchiness to both abundance and occupancy, as exemplified by seagrass macrobenthos.**

R.S.K. Barnes

*Department* *of Zoology and Entomology, Rhodes University, Makhanda, Eastern Cape, Republic of South Africa*

*School of Biological Sciences & Centre for Marine Science, University of Queensland, Brisbane, Queensland, Australia*

*Department of Zoology & Conservation Research Institute, University of Cambridge, Cambridge, UK*

Author's email: rsb1001@cam.ac.uk

**1: Location of samples.**

Datasets of the macrobenthic seagrass assemblage components from Moreton Bay were those assembled by Barnes (2020), together with those from peripheral assemblages near the margin of the Deanbilla and Goompi seagrass beds with adjacent bare sand (Barnes and Hamylton, 2013, 2016) and with mangrove at Deanbilla (Barnes, 2017). All samples were located between MLW and LWS along a 7 km stretch of coast from Deanbilla (27º30'33"S,153º24'36"E) to Capembah (27º27'54"S,153º25'22"E). Equivalent datasets from the Knysna estuarine bay were the intertidal ones assembled by Barnes (2019a,b), plus the sublittoral datasets of Barnes and Claassens (2020), and they extended along the main axial channel of the bay from the mouth region at Bollard Bay (34º04'01"S,23º03'09"E) to near the head at Red Bridge (34º00'54"S,23º00'14"E), a distance of some 12 km along the estuarine channel but 7 km in a direct line. Data on the mean abundance, patchiness and occupancy for each of these species at each site at which they were present in any numbers (see article) at the two localities are given in Electronic Supplementary Information 2.

Barnes RSK 2017. Are seaward pneumatophore fringes transitional between mangrove and lower-shore system compartments? Mar Environ Res 125:99-109.

Barnes RSK 2019a. Abundance/occupancy/patchiness relations in estuarine seagrass macrobenthos. Estuar Coast Shelf Sci 228:106360.

Barnes RSK 2019b. Local patchiness of macrobenthic faunal abundance displays homogeneity across the disparate seagrass systems of an estuarine bay. Mar Environ Res 148:99-107.

Barnes RSK 2020. Do species display characteristic intraspecific levels of patchiness in a given habitat type? The case of intertidal seagrass macrobenthos. Mar Biol 167:177.

Barnes RSK, Claassens L 2020. Do beds of subtidal estuarine seagrass constitute a refuge for macrobenthic biodiversity threatened intertidally? Biodivers Conserv 29:3227-3244.

Barnes RSK, Hamylton S 2013. Abrupt transitions between macrobenthic faunal assemblages across seagrass bed margins. Estuar Coast Shelf Sci 131:213-223.

Barnes RSK, Hamylton S 2016. On the very edge: faunal and functional responses to the interface between benthic seagrass and unvegetated-sand assemblages. Mar Ecol Progr Ser 553:33-48.


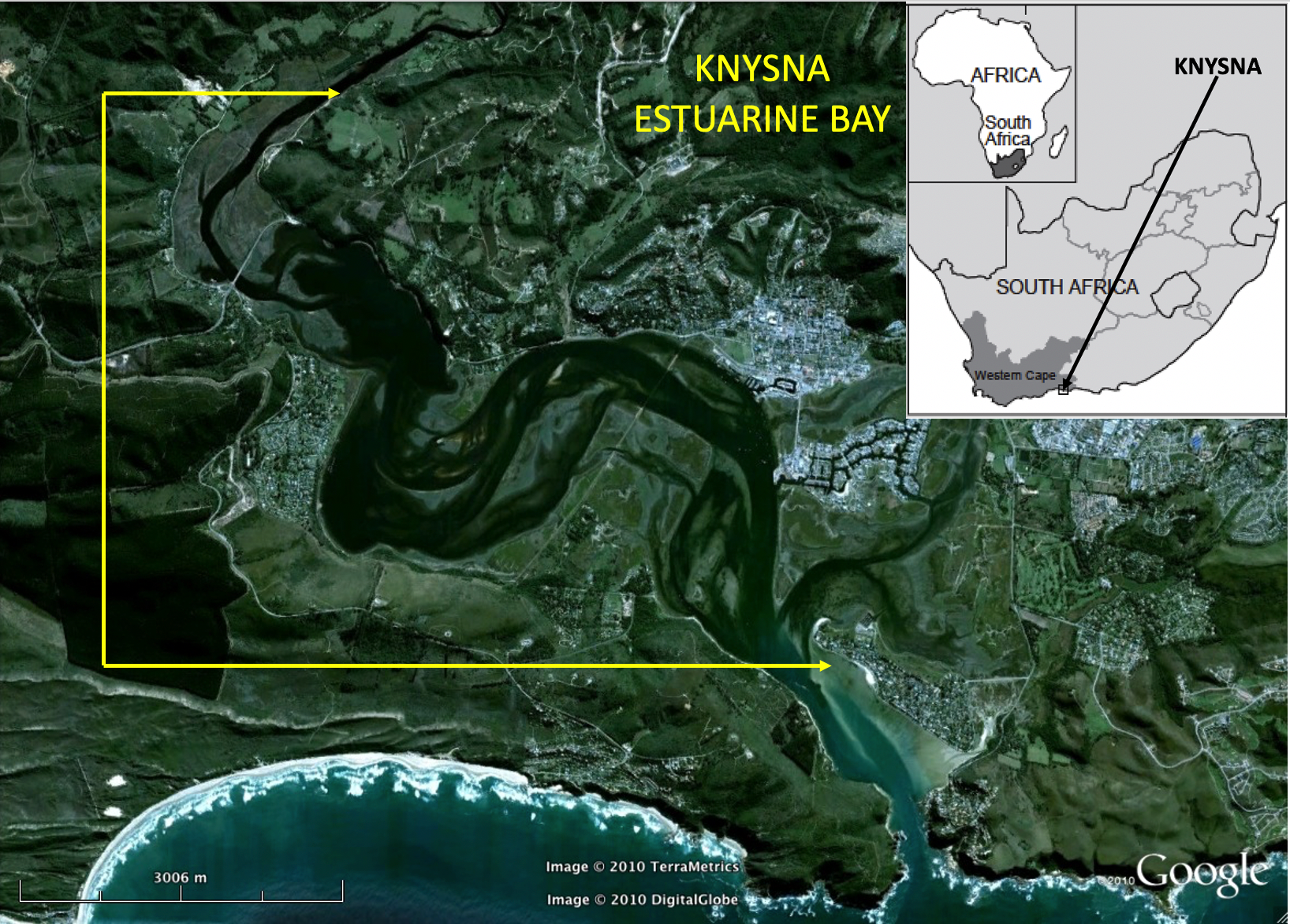


Google Earth image of Knysna Estuarine Bay - all datasets located between the two arrows.


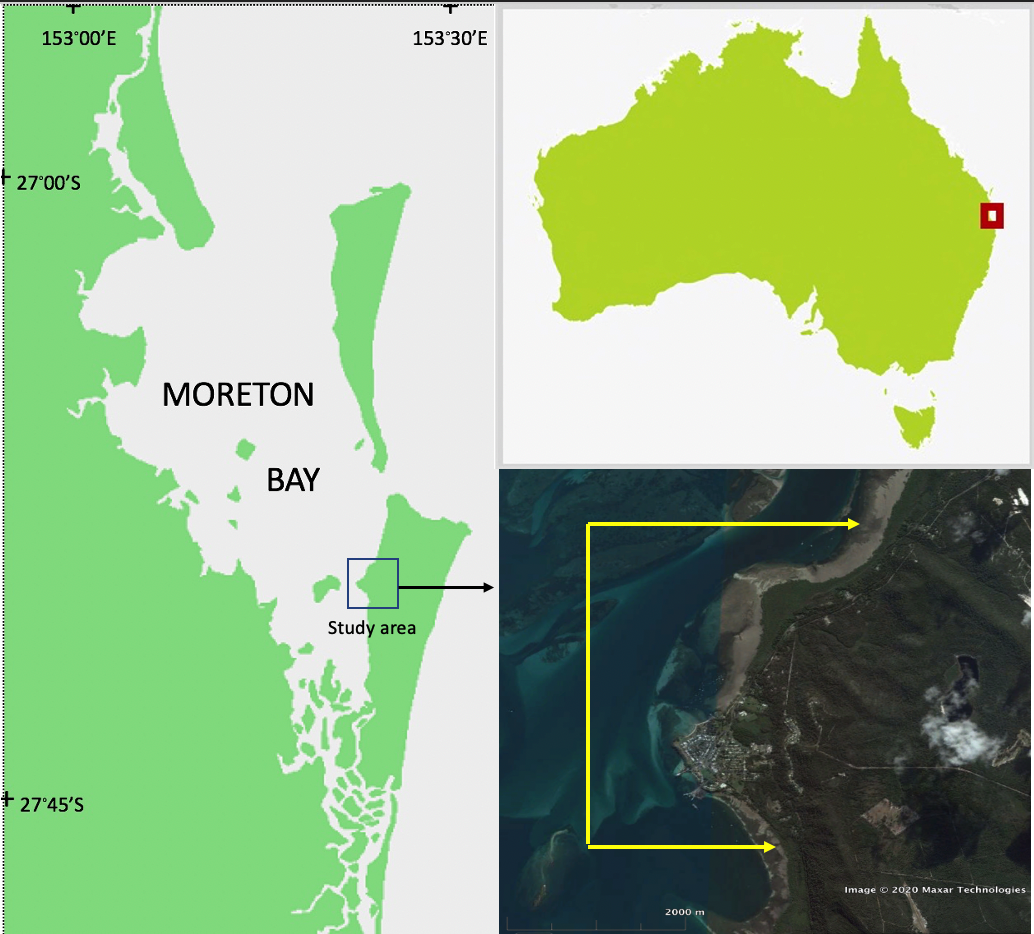


Location of Moreton Bay samples on the northwest coast of North Stradbroke Island - all datasets collected between the two arrows.
